# Supporting Lung Cancer Patients With an Interactive Patient Portal: Feasibility Study

**DOI:** 10.2196/cancer.7443

**Published:** 2017-08-08

**Authors:** Wim G Groen, Wilma Kuijpers, Hester SA Oldenburg, Michel WJM Wouters, Neil K Aaronson, Wim H van Harten

**Affiliations:** ^1^ Division of Psychosocial Research and Epidemiology The Netherlands Cancer Institute Amsterdam Netherlands; ^2^ Division of Surgical Oncology The Netherlands Cancer Institute Amsterdam Netherlands; ^3^ Department of Health Technology and Services Research University of Twente Enschede Netherlands; ^4^ Rijnstate Hospital Arnhem Netherlands

**Keywords:** non-small cell lung cancer, patient empowerment, patient portal, supportive care, eHealth, feasibility

## Abstract

**Background:**

MyAVL is an interactive portal for cancer patients that aims to support lung cancer patients.

**Objective:**

We aimed to evaluate the feasibility and usability of the patient portal and generate preliminary evidence on its impact.

**Methods:**

Lung cancer patients currently or recently treated with curative intent could use MyAVL noncommittally for 4 months. Feasibility, usability, and preliminary impact (ie, patient activation, quality of life, and physical activity) were studied by means of questionnaires, a focus group, and analysis of user log data.

**Results:**

We included 37 of 123 eligible patients (mean age 59.6 years). The majority of responses (82%) were positive about using MyAVL, 69% saw it as a valuable addition to care, and 56% perceived increased control over their health. No positive effects could be substantiated on the impact measures.

**Conclusions:**

MyAVL appears to be a feasible and user-friendly, multifunctional eHealth program for a selected group of lung cancer patients. However, it needs further improvements to positively impact patient outcomes.

## Introduction

Cancer and its treatment result in a wide range of physical and psychological challenges, some of which may appear years later [1], and current models of survivorship care may not be sustainable [2]. Therefore it seems imperative that cancer survivors play a more active role in their health care. One way to support this active role may be by enhancing their levels of empowerment, which encompasses being autonomous and having the knowledge and psychosocial and behavioral skills to influence one’s health in a positive way [3]. eHealth programs may be helpful to support aspects of patient empowerment in individuals with chronic diseases and also cancer survivors [3,4]. eHealth programs can improve aspects of empowerment by enhancing patients’ knowledge of their disease and treatments and about their own health status (eg, via patient-reported outcomes [PROs]) [3].

To date, many eHealth services in oncology have been developed for breast and prostate cancer patients [5]. Although lung cancer has a high symptom burden, very few eHealth applications have been developed recently to support this patient population, mainly related to symptom monitoring [6-11]. To support lung cancer patients in the Netherlands Cancer Institute Antoni van Leeuwenhoek Hospital (AVL in Dutch), we developed an interactive portal (MijnAVL; MyAVL in English). MyAVL includes patient education, an overview of appointments, access to the electronic medical record (EMR), PROs with feedback of the scores, and tailored physical activity support. We developed MyAVL and selected its most relevant features following a stepwise approach: literature review [4], focus groups with patients and health professionals [12], acceptability testing based on mock-ups, and usability testing of functional prototypes [13].

The aim of this study was to evaluate MyAVL’s feasibility and usability and to generate preliminary evidence on its impact when used by lung cancer patients.

## Methods

### Patients and Recruitment

We included patients with non-small cell lung cancer who were currently being treated or who had completed primary, curative treatment up to 12 months earlier. Treatments included surgery, radiotherapy, concurrent chemoradiotherapy, or a combination of these. Patients were approached by letter followed by a phone call from the researchers to discuss participation and check further eligibility criteria (eg, having a computer and Internet access, mastery of the Dutch language). Patients provided written informed consent, and the study procedures were approved by the local Institutional Review Board. Because the primary aim of the study was to test feasibility and usability of the portal, no a priori power calculation was performed and as many patients as possible were recruited within the project timeline.

### MyAVL Intervention

**Figure 1:**
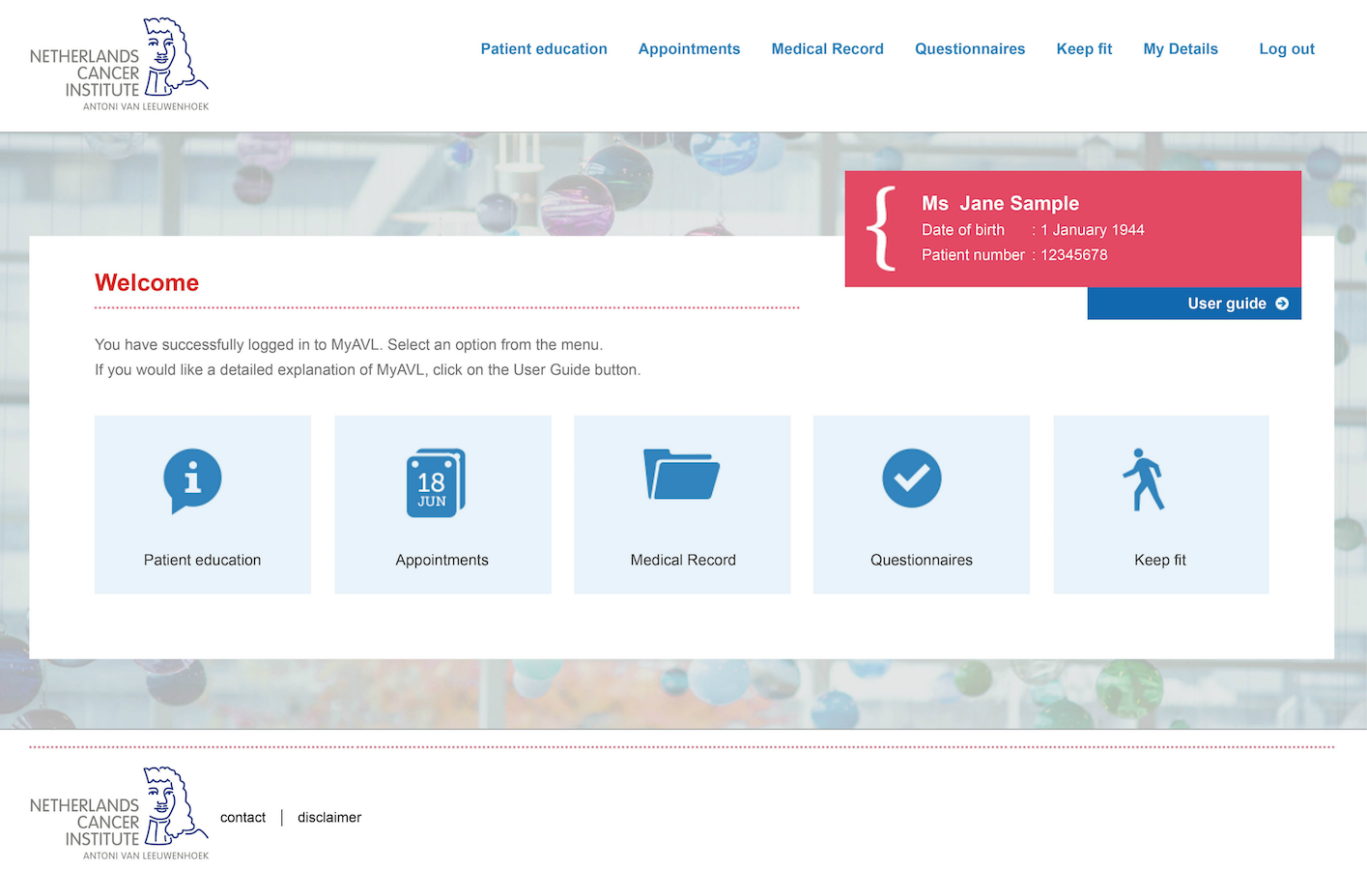
Homepage of MyAVL.

The content of MyAVL, including screenshots of its features, have been described in detail previously [13,14]. In short, it includes 5 features: (1) personalized patient education material (health professionals provide the most timely and suitable patient education materials); (2) an overview of past and upcoming appointments; (3) access to the EMR, including blood tests, physiological test results (eg, lung function), pathology reports, and letters to the general practitioner and other hospitals (with medical test results made available with a 2-week delay); (4) PROs and related feedback (ie, a graphical and tabular overview of scores and access to background information on quality of life aspects such as fatigue); and (5) tailored physical activity advice based on a set of questionnaires assessing physical activity levels, motivation, and possible contraindications. MyAVL could be used noncommittally for 4 months, meaning that patients did not have to adhere to a predefined intervention schedule. Figure 1 shows a screenshot of the homepage of MyAVL.

### Assessments

At baseline, participants completed questionnaires on sociodemographic and effect measures: patient activation (Patient Activitation Measure [PAM]) [15-17], quality of life (Short Form Health Survey [SF-36]) [18], and physical activity (International Physical Activity Questionnaire [IPAQ]) [19]. After 4 months, log data on actual use were analyzed retrospectively, and participants completed questions on self-reported use, satisfaction (Website User Satisfaction questionnaire [WUS]), acceptability (a questionnaire based on the unified theory of acceptance and use of technology [UTAUT]) [20], and the effect measures PAM, SF-36, and IPAQ. Physical activity was expressed as metabolic equivalent of task (MET) minutes per week for moderate, vigorous, and total activity. To evaluate acceptability per component of the portal, questions were posed on aspects like level of personalization, level of comprehensibility, and level of anxiety. The response scale of these questions ranged from strongly disagree to strongly agree. Patients also rated the different components on a scale from 1 to 10 (higher scores being more positive ratings). Finally, a focus group was held with 5 participants to further discuss the pros and cons of using MyAVL and its features. The content of the focus group discussion was structured around issues that arose on the questionnaires. The session was audiorecorded, and notes were taken.

### Analyses

Data on feasibility (eg, use) and acceptability were analyzed with descriptive statistics. Data on the PAM and SF-36 were presented as means and standard deviations, the IPAQ as median and interquartile range. The PAM, SF-36, and IPAQ questionnaires were scored according to standard scoring procedures. Pre- and posttest scores were compared by a paired samples *t* test except for the IPAQ, which was tested with the related samples Wilcoxon signed rank test. Focus group data were analyzed by the first author reviewing the notes and integrating these findings with the open-ended evaluative questions of the postintervention questionnaire. Topics were included if they were raised by at least 2 patients. The second author validated the formation of topics from the data. Patients needed to log in at least once to be included in the analyses. Statistical analyses were performed with SPSS version 22 (IBM Corp).

## Results

### Feasibility

Between January 2014 and August 2015, 123 patients were eligible for the study, 89 of these could be reached and were asked to participate, and 37 agreed to do so. The most common reasons for declining were having little computer or Internet experience (n=14), emotionally too burdensome (n=12), and not having a computer or Internet access (n=9).

All patients were white, and 16/34 were women (47%). Mean age of the subjects was 59.6 (SD 8.4, range 40-76) years. The majority of patients were in a relationship with someone whom they lived with and had completed postcompulsory education, and 27/34 patients (79%) were in treatment. Sociodemographic and clinical characteristics are presented in Table 1. Nearly all patients (33/34, 97%) had used the Internet more than 3 years, and 31/34 (91%) used it (almost) daily.

The mean number of log-ins during the 4 month study period was 11.2 (SD 9.1, range 0-30) with a mean duration of 12.9 (SD 13.9, range 1-77) minutes. A total of 3 patients did not log in at all and were not included in further analyses. Overview of appointments, access to EMR, and questionnaires were used most frequently, with an average of 7.5 (SD 7.0), 6.7 (SD 4.7), and 6.7 (SD 5.0) log-ins, respectively. The remaining components, patient education, quality of life scores, and Keep Fit, were accessed less often, with an average of 1.9 (SD 2.4), 3.7 (SD 3.1), and 3.1 (SD 2.5) views, respectively. On average, 2.3 (SD 2.5) PROs were completed, which is 82% of total number of PROs provided (SD 36%). The mean number of Keep Fit questionnaires filled out was 2.0 (SD 1.3). Males more frequently than females accessed overview of appointments (9.6 [SD 6.6] vs 4.6 [SD 6.6], *P*=.04) and the questionnaires section (8.4 [SD 5.3] vs 3.9 [SD 3.0], *P*<.01). No significant differences between male and female participants were noted for the other components of the portal or for the total number of log-ins. No significant differences in these variables were noted between patients in and out of treatment.

### Usability

Acceptability data, as measured with the UTAUT-based questionnaire, indicated that 93% (25/27) of patients found MyAVL easy to use, 56% (15/27) reported that it contributed to a sense of control over their health, and 69% (18/26) indicated that it was a valuable addition to their health care experience. Most (22/27, 81%) were satisfied with MyAVL, and 77% (20/26) intended to continue using it. A total of 61% (17/28) reported being better informed about their disease via access to the EMR, and 43% (12/28) reported an enhanced sense of control over their disease. Average satisfaction rating (WUS score) across domains was 3.9 (maximum score is 5). Key issues that emerged from the acceptability questions and focus group are presented in Table 2.

### Preliminary Data on Impact

PAM scores actually decreased slightly over time from 64.8 (SD 14.2) to 59.4 (SD 11.6) (*P*=.042). For the SF-36, we found no significant changes over time. Levels of physical activity did not change significantly, but vigorous physical activity tended to increase over time from a median of 0 (interquartile range, [IQR] 0-840) to 240 (IQR 0-1140) MET minutes per week (*P*=.053).

**Table 1:** Patient characteristics.

Characteristic		Total
Sex (female), n (%)		16 (47)
Age, years, mean (SD)		59.6 (8.4)
**Marital status, n (%)**		
	Relationship, married, living together	26 (76)
	Divorced	4 (12)
	Widowed	3 (9)
	Missing	1 (3)
**Education, n (%)**		
	Compulsory or less	2 (6)
	Postcompulsory	21 (62)
	University or college	9 (26)
	Other	2 (6)
**Employment status, n (%)**		
	Full-time job	11 (32)
	Part-time job	3(9)
	Homemaker	1(3)
	Retired	11(32)
	Volunteer worker	1 (3)
	Disabled	5 (15)
	Missing	2 (6)
**Cancer stage, n (%)**		
	I	13 (38)
	II	5 (15)
	III	16 (47)
**Type of treatment, n (%)**		
	Surgery only	12 (35)
	Surgery and chemotherapy	3 (9)
	Concurrent chemoradiotherapy only	10 (29)
	Concurrent chemoradiotherapy and surgery	2 (6)
	Radiotherapy only	7 (21)
Currently in treatment, n (%)		27 (79)
Comordity present, n (%)		22 (65)

**Table 2:** Acceptability of MyAVL as a whole and its components.

MyAVL component	Used this feature (self-report) N=28 n (%)	Rating (1-10) mean (SD)	Key remarks, issues, and suggestions for improvement based on questionnaire and focus group data
MyAVL as a whole	—	7.8 (0.9)	The 2-step authorization procedure (with username, password, and text message authentication) was found to be burdensome Issues with non-Windows operating system (ie, iOS) Some patients indicated that they logged in to the program less frequently because they had noted that the content of the portal did not change much during the course of the study
Patient education	11 (37)	7.1 (1.5)	Patients indicated that too few documents were available Some indicated that content could be more tailored to specific complaints of patients
Appointments	25 (83)	8.2 (1.2)	No major issues; very comprehensible and useful Past appointments were found to be useful for reimbursement purposes
Access to the EMR^a^	24 (80)	7.1 (1.1)	Although many patients found the information useful and comprehensible, it also raised questions or anxiety in some cases Not all data of the EMR could be accessed. Some wanted to see more (eg, imaging results, doctors’ personal notes). The delay of 2 weeks before showing test results was perceived as too long by some patients. The delay should be indicated more clearly in the portal. Data from other hospitals could not be seen via MyAVL
PROs^b^and feedback	21 (70)	7.4 (1.0)	Graphs and tables with scores were comprehensible and valued by patients as these gave insight into their quality of life over time Some indicated that PROs were somewhat unpleasant to complete or took too much time to complete PROs were not often discussed during medical consultations, which disappointed some patients
Keep Fit	8 (27)	7.2 (0.8)	Reminded several patients of the importance of physical activity Advice was sometimes perceived as too general and could be more tailored Recalling the amount of physical activity during the past week (needed for the questionnaire) was not always easy Some expressed desire for a free text option to express their concerns or needs in this respect Some expressed the need for information on services (eg, physiotherapy) that are specialized for cancer patients

^a^EMR: electronic medical record.

^b^PROs: patient-reported outcomes.

## Discussion

MyAVL, an eHealth program developed in an oncology setting, was found to be feasible, easy to use, and useful by the majority of the lung cancer patients who participated in the study. Access to the EMR and the overview of appointments were evaluated very positively and used quite frequently. We expected positive effects of access to the EMR in terms of improved knowledge, autonomy, self-efficacy, and patient-clinician communication [21]. Our results supported this in part: 61% (17/28) reported that this information enhanced knowledge of their disease and 43% (12/28) indicated that it enhanced their sense of control over their disease. In general, patients indicated that they would prefer access to their full medical record and access to medical test results as soon as they have been reviewed by a professional. Reassuringly and similar to other studies [22,23], very few patients reported that having access to the EMR led to feelings of (mild) anxiety. At the same time, our measures of impact (ie, PAM, SF-36, and IPAQ) indicated no improvement over time. In fact, there was a significant, albeit small, decrease in PAM scores. One explanation may be that these outcome measures are not responsive enough to the possible effects of the portal or that the “dose” was not strong enough. For future trials on these types of interventions, more tailored or specific outcome measures may be needed. The supporting effects of MyAVL (and patient portals in general) may be further increased by adding features focused specifically on coping and symptom control [3].

eHealth in lung cancer patients is a relatively new occurence, and few studies have been published. Most of these studies are related to symptom monitoring [7,8,10,11], which is very different from our multicomponent intervention. One study by Gustafson et al [9] reported the results of a trial in which they compared the use of a comprehensive online intervention (Comprehensive Health Enhancement Support System [CHESS]) with standard Internet access in palliative lung cancer patients and especially their caregivers. CHESS included information, communication with and support from peers, coaching feedback based on user input, and tools to organize support from family and friends. The researchers found that caregivers in the CHESS arm consistently reported lower patient physical symptom distress than caregivers in the Internet arm. Unfortunately, we did not measure symptom distress in our study so we cannot compare our findings on this aspect. The actual use they reported was quite low, with only 73.4% of caregivers and 50% of patients accessing CHESS at least once. In contrast, in our study, 34/37 patients (92%) used the application more than once. This higher use may be related to our patient sample as we only included patients who were treated with curative intent. These patients may be more capable or willing to use supporting eHealth programs than patients who receive palliative treatment. Median length of use in the Gustafson study was 103 minutes for caregivers and 146 minutes for patients, compared to a mean log-in time of 12.9 minutes in our study. This large difference might be related to the broader range of supporting tools included in CHESS.

Difficulty with patient accrual appears to be a common theme among eHealth studies in lung cancer patients [9,11]. We are not aware of any direct comparative data on interest in or use of eHealth by different cancer patient populations. However, in our study, the participation rate of patients who could be contacted was 42%, whereas in a previous study of breast cancer patients the participation rate was higher (52%) [14]. One could thus argue that lung cancer patients may be less willing to participate in such interventions. Several previous studies, including Gustafson et al [9] and Cleeland et al [11], recruited fewer patients than planned. On a positive note, those patients who did participate in our study were, in general, very satisfied with the portal. Thus for interested and motivated patients, such eHealth approaches may be very suitable.

Despite the large potential benefits of exercise [24], the physical activity support program was used by only one-third of participants. Those who received intensive treatment (eg, chemoradiotherapy) were particularly unlikely to use the program. This may be an indicator of limited feasibility of this part of the portal for these patients.

We observed relatively good compliance with completing PROs during the study period, which may be due to the fact that the PROs are perceived as part of their integrated care [25]. The accessibility of MyAVL may be further enhanced by simplifying the authorization/access procedure (Table 2).

A clear limitation of this study is the low participation rate and resulting small sample size. This small and select sample of patients may limit the generalizability of our findings, as participating patients may differ from the majority of lung cancer patients. Additionally, several components of the portal (eg, the Keep Fit component) were not used by every patient, which led to evaluations of these components by a relatively small number of patients. This might indicate that these components are less feasible for lung cancer patients. The limited number of patients in the focus group may not fully represent the views of the total group of patients. A final limitation is that patient knowledge of their disease was measured by self-report and not measured objectively, which may be subject to bias.

In conclusion, MyAVL appears to be a feasible and user-friendly multifunctional eHealth program for patients with lung cancer, although participation rate was quite low. Additional efforts are needed to increase the reach and effect of the program in terms of patient empowerment and to increase the attractiveness, perceived value, and use of the patient education and physical exercise elements of the program.

## Abbreviations

AVL: Antoni van Leeuwenhoek
CHESS: Comprehensive Health Enhancement Support System
EMR: electronic medical record
IPAQ: international Physical Activity Questionnaire
IQR: interquartile range
PAM: Patient Activation Measure
MET: metabolic equivalent of task
SF-36: Short Form Health Survey
PRO: patient-reported outcome
UTAUT: unified theory of acceptance and use of technology
WUS: Website User Satisfaction questionnaire
